# Proof of Concept for Open Flow Microperfusion for Intratumoural Drug Administration in Equine Cutaneous Neoplasia

**DOI:** 10.3390/ani16193090

**Published:** 2026-10-01

**Authors:** Raphael Labens, Paul Reddell, Thomas Birngruber

**Affiliations:** 1 School of Agricultural, Environmental and Veterinary Sciences, Charles Sturt University, Wagga Wagga, NSW 2678, Australia; 2 QBiotics Group Limited, Brisbane, QLD 4066, Australia; paul.reddell@qbiotics.com; 3 HEALTH—Institute for Biomedical Research and Technologies, Joanneum Research, 8010 Graz, Austria; thomas.birngruber@joanneum.at

**Keywords:** tigilanol tiglate, implant, perfusion, sarcoid, horse

## Abstract

Intratumoural drug delivery can achieve high local therapeutic exposure while limiting systemic effects, but heterogeneous distribution may compromise treatment efficacy and precision. This proof-of-concept study investigated open flow microperfusion (OFM) as a novel platform for local delivery of tigilanol tiglate (TT), an intratumoural antineoplastic agent with immunomodulatory and vascular-disrupting effects. Four horses with histologically confirmed cutaneous neoplasia (three sarcoids and one squamous cell carcinoma) underwent OFM using TT concentrations of 0.01–0.04 mg/mL delivered at 9 µL/min for 7–24 h over one to three consecutive days. OFM systems were well tolerated and remained functional for up to three days. Localized vascular disruption, polymorphonuclear cell recruitment, oncosis and tissue sloughing developed in association with perfused regions, consistent with recognized TT-induced tissue responses. Two sarcoids exhibited biologic responses that did not result in complete local tumour ablation. Tumour ablation was achieved initially in one squamous cell carcinoma, although deep-seated disease subsequently progressed, while one sarcoid was ablated and remained tumour-free 497 days after treatment. These preliminary findings demonstrate that OFM allows precise, sustained intratumoural TT challenge, resulting in characteristic tissue responses supporting further investigation of this local delivery platform for tumour-targeted therapies.

## Simple Summary

This study tested a new way of delivering an anti-cancer drug, tigilanol tiglate, directly into tumours using a technique called open flow microperfusion. The aim was to deliver the drug precisely and continuously to the tumour while reducing exposure to the rest of the body. Four horses with skin tumours were treated. The perfusion system remained functional for up to three days and was generally well tolerated by the animal. Treatment caused expected anti-tumour effects around the delivery sites, including damage to tumour blood vessels, recruitment of immune cells, tumour cell death, and tissue sloughing. One tumour was initially completely destroyed, although deeper cancer later progressed. Another horse remained free of its treated tumour long term. This study provides early evidence that this system could be a useful method for delivering cancer drugs directly and precisely into tumours. However, as only four horses were studied, further research is needed to determine how effective and reliable this approach is.

## 1. Introduction

Intratumoural administration represents an important therapeutic route for the management of equine cutaneous neoplasia [1]. This approach is partly driven by the adverse effects, costs, and practical limitations associated with systemic chemotherapy [1], while some therapeutic strategies specifically depend on local immunogenic responses for efficacy [2].

Tigilanol tiglate (TT), a naturally occurring epoxytigliane diterpene ester derived from the Australian blushwood tree (*Fontainea picrosperma*), has demonstrated efficacy against a range of cutaneous neoplasms [3,4,5,6,7]. Its local effects are characterized by vascular disruption, inflammatory and immune cell recruitment, tumour cell oncosis, and subsequent tissue necrosis and sloughing [8,9]. Although TT was developed for a single intratumoural injection, biologically relevant effects were also observed using repeated low-dose and slow-release delivery strategies, directed specifically at the tumour base [10]. These observations are particularly relevant given the potential for heterogeneous drug distribution following intratumoural injection, especially in dense, poorly permeable, or highly ulcerated tumours where leakage may limit effective intratumoural exposure.

Open flow microperfusion (OFM) is a minimally invasive, membrane-free in vivo technique that enables continuous exchange with, and sampling of, interstitial fluid from a defined tissue compartment. In OFM, a miniaturised low-flow peristaltic pump circulates perfusate through an implanted PEEK-mesh linear probe. The small fenestrations permit bidirectional exchange between the perfusate and surrounding interstitial fluid through diffusion and convection, producing a diluted but unfiltered interstitial fluid sample [11]. Although OFM is well established for tissue sampling and dermal pharmacokinetic studies and has more recently been explored for characterisation and sensing of the tumour microenvironment, its potential as a primary local drug delivery platform has not yet been investigated [12,13]. Given the aforementioned limitations, OFM may facilitate therapeutic delivery in dense tumours and minimize therapeutic leakage from target tissues. Furthermore, in contrast to slow/delayed release systems currently in clinical use [14], OFM may enable both temporal and spatial control over chemotherapeutic delivery.

Here, we aimed to provide proof of concept for the technical feasibility of OFM as a local delivery platform for TT and to characterise the resulting tissue responses associated with low-dose TT exposure. We hypothesised that continuous, low-flow administration of low-dose TT via OFM would induce the characteristic tissue responses associated with intratumoural TT treatment, including inflammatory and immune cell recruitment, tumour cell oncosis, and subsequent tumour ablation.

## 2. Materials and Methods

Animals/Tumours: Four horses with cutaneous neoplasia and histopathologic confirmation (three sarcoids and one squamous cell carcinoma) were enrolled under caretakers’ consent and institutional animal care and ethics committee approval. Case selection was opportunistic and governed by clinician and caretaker schedules to allow hospitalization and treatment. Caretakers acknowledged the exploratory nature of the treatment approach and accepted the study sponsor’s financial support (QBiotics Group Limited, Brisbane, QLD, Australia). In two cases, OFM was performed in conjunction with other treatment interventions (TT eluting implant; autotransplantation of sarcoid tissue), while in the remainder, OFM with TT represented the only treatment modality. Tumour volume (T_Vol_; cm^3^) was calculated based on the following formula: *Tumour width × length × depth* (cm) × 0.5.

OFM settings: In all instances OFM was performed with the perfusion system in the push as opposed to the push–pull configuration [13]. This meant that only one peristaltic pump head was used to propel perfusate in the system, rather than a second being connected to the egress line and concurrently pulling perfusate from the target area. Systems were perfused with a 0.01–0.04 mg/mL TT solution prepared by diluting a 0.1% aqueous mixture of TT in water and propylene glycol (60/40% *v*/*v*) with 0.9% NaCl. OFM was performed for variable durations (see below) utilizing a 9 μL/min flow rate of the TT solution. At most, perfusions were performed for 8 h to begin and finish on the same day and allow an overnight break in case they were recommenced the next day.

**Figure 1:**
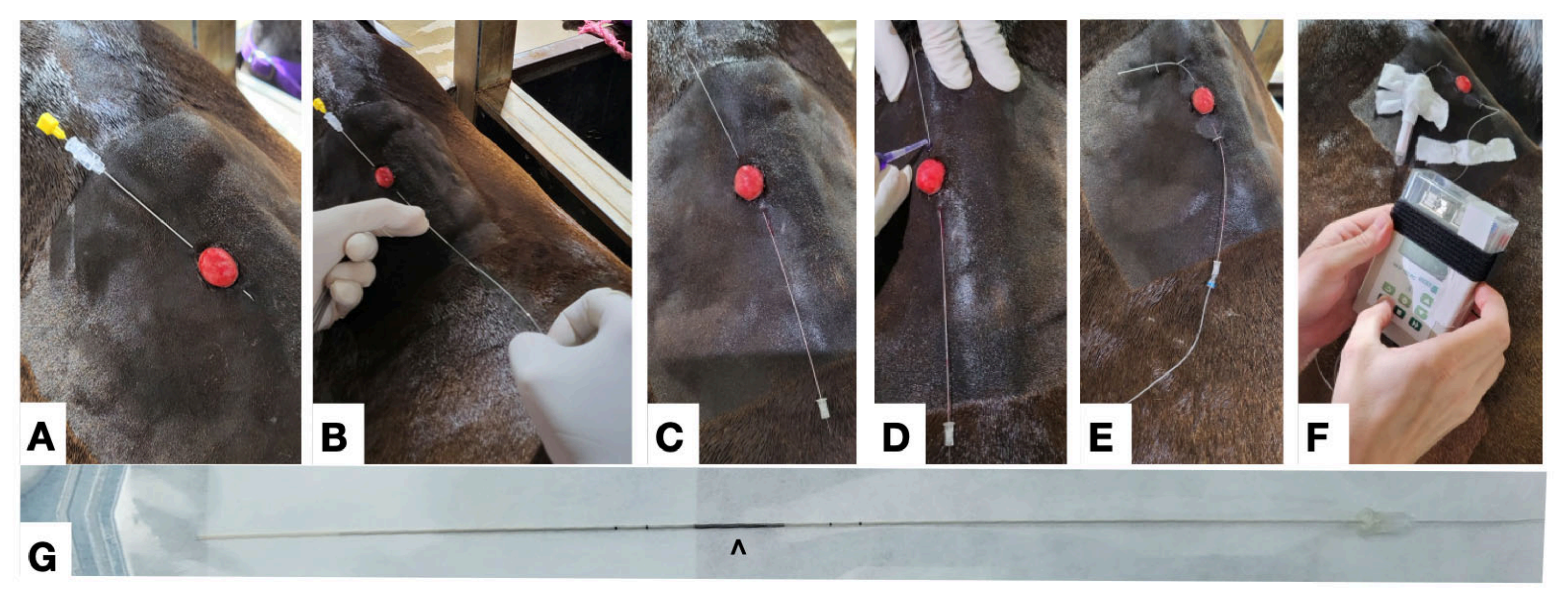
System set-up. An open flow perfusion probe with a central fluid exchange window (black arrowhead; (**G**)) was threaded in retrograde fashion through a 21 G, 3.5-inch-long needle (**B**), preplaced through the base of the tumour (**A**), with the objective to position the fluid exchange window of the probe in the tumour base (**C**). Sites were sealed using skin glue (**D**), probes connected to the peristaltic pump, and the perfusion commenced in an open configuration (**E**,**F**).

Systems install: Under intravenous sedation (detomidine; 0.01 mg/kg IV; butorphanol; 0.01 mg/kg IV), target areas were clipped and aseptically prepared (4% chlorhexidine gluconate or 10% povidone-iodine antiseptic scrub followed by 70% isopropyl wipe) and locoregional analgesia performed (2% mepivacaine hydrochloride solution). Figure 1 illustrates the steps associated with the system set-up. An open flow perfusion probe (diameter 0.55 mm) with a variably sized fluid exchange window containing 0.2 mm fenestrations (black arrowhead; Figure 1G) was threaded in retrograde fashion through a 21 G, 3.5-inch-long needle (Figure 1B), preplaced through the base of the tumour (Figure 1A). Probe entry and exit points were located approximately 5–10 mm from the tumour margin (Figure 1C), with the objective to position the fluid exchange window in the tumour base. Sites were sealed using medical skin glue (Figure 1D), lines connected to the peristaltic pump, and the perfusion commenced in an open configuration, allowing egress to free flow into a separately affixed sample tube (Figure 1F). Depending on the treatment site, the pump was either affixed to the horse’s head collar or maintained in a dedicated belt positioned around the horse’s girth. While in operation, systems were monitored every 30 min for continuous flow exiting the probe, but effluent volume was not quantified.

Concurrent medications: All horses received flunixin meglumine (1.1 mg/kg IV) and dexamethasone (0.1 mg/kg IV) once every treatment day and thereafter were continued on non-steroidal medication as needed (phenylbutazone; 2.2 mg/kg PO, SID–BID).

### 2.1. Exploratory Outcome Assessments/Follow-Up

Animals remained in hospital care after completion of perfusions until TT-related acute inflammatory responses had subsided and oncosis patterns were established, typically occurring between four and six days post treatment. During this time, twice daily physical examinations included evaluation of pain (reaction to palpation), tumour tissue swelling and tissue viability. In one horse, thermographic imaging was used to monitor skin responses.

Dye distribution on trimmed tumour surfaces and developing tumour necrosis margins were measured using digital calipers. Once patients were discharged, weekly photographic updates by caretakers informed on progress. Horse 4 was housed in close proximity, allowing weekly in-person follow-up by the first author.

Fresh histopathologic samples taken following completion of final perfusions were first visually assessed for dye distribution and then, following further processing, microscopically for TT-associated changes. Polymorphonucleated cell influx and vascular disruption (extravascular interstitial erythrocytes) were assessed as present/absent. Dye distribution was subjectively compared to areas of TT histopathological changes by the first author who was not blinded to treatments.

### 2.2. Details Specific to Each Enrolled Patient

**Figure 2:**
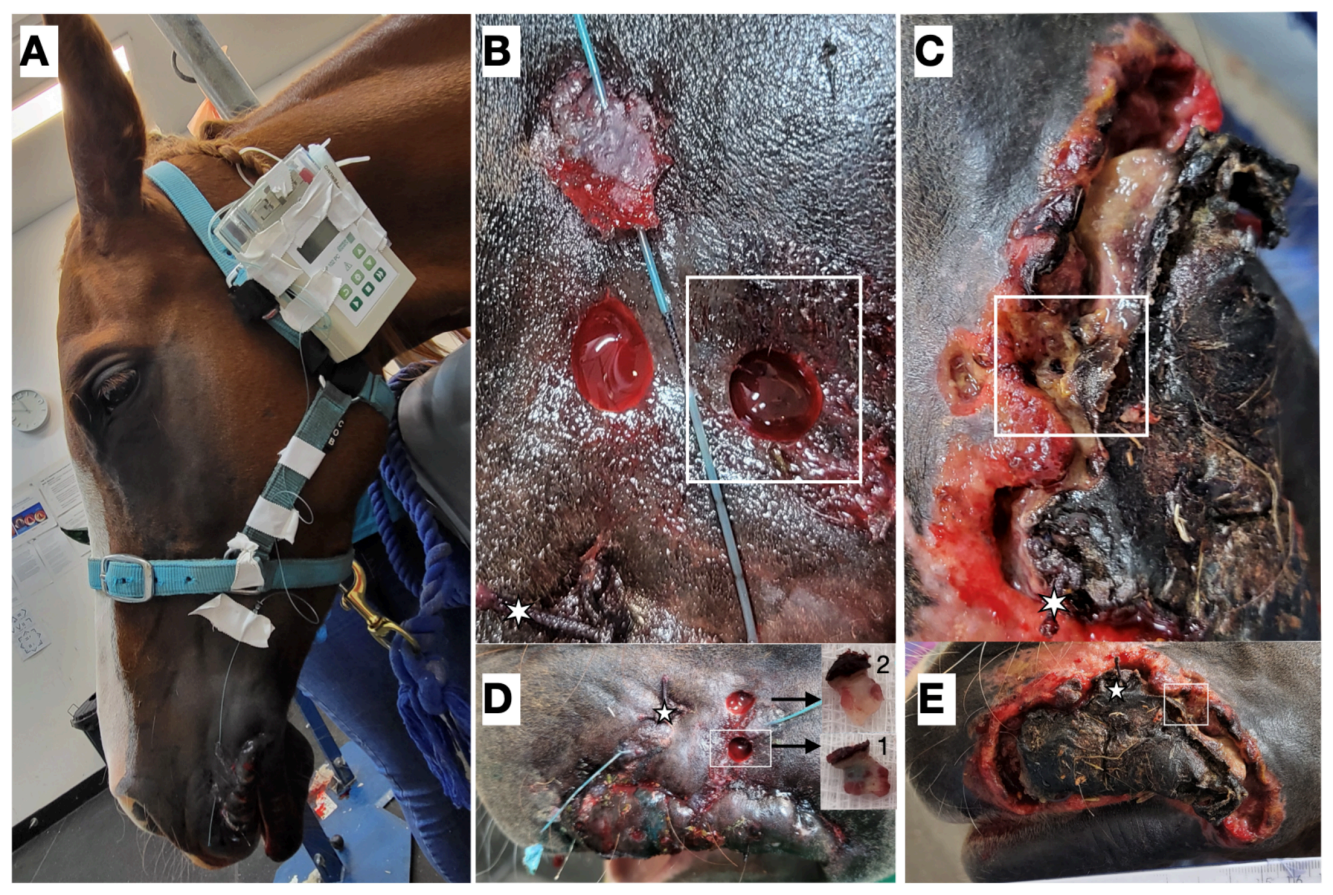
Open flow microperfusion of occult sarcoid tissue (Horse 1). System set-up (**A**), tissue appearance at conclusion of perfusion (**B**), and after 5 days (**C**). In (**B**) the perfusing probe has been removed and laid onto the skin to illustrate its previous position relative to the sampling sites. The blue indicator dye is seen in the ventral ((**1**); (**D**)) but not the dorsal ((**2**); (**D**)) tissue biopsy sample and the dark appearance of its sampling site is consistent with early TT-associated tissue changes. Five days later implant-induced oncosis and tissue slough is apparent. Tissue slough extends into the pump-associated treatment area and the ventral sampling site (white box, **B**–**E**). An adjacent therapeutic implant (white star) is highlighted for orientation.

Horse 1 (7-year-old Arab mare)—While the primary tumour, a fibroblastic sarcoid (2 × 6 × 1.5 cm; T_Vol_ 9 cm^3^) involving the left side of the upper lip, was treated with an eluting implant, a small test area in the dorsally adjacent occult sarcoid tissue, underwent 7 h of OFM with blue dye added to the TT solution (0.04 mg/mL). Upon completion of OFM, a 6 mm skin punch biopsy was obtained on either side of the 5 mm long fluid exchange window (Figure 2) and the exposed tissues inspected for discoloration.

**Figure 3:**
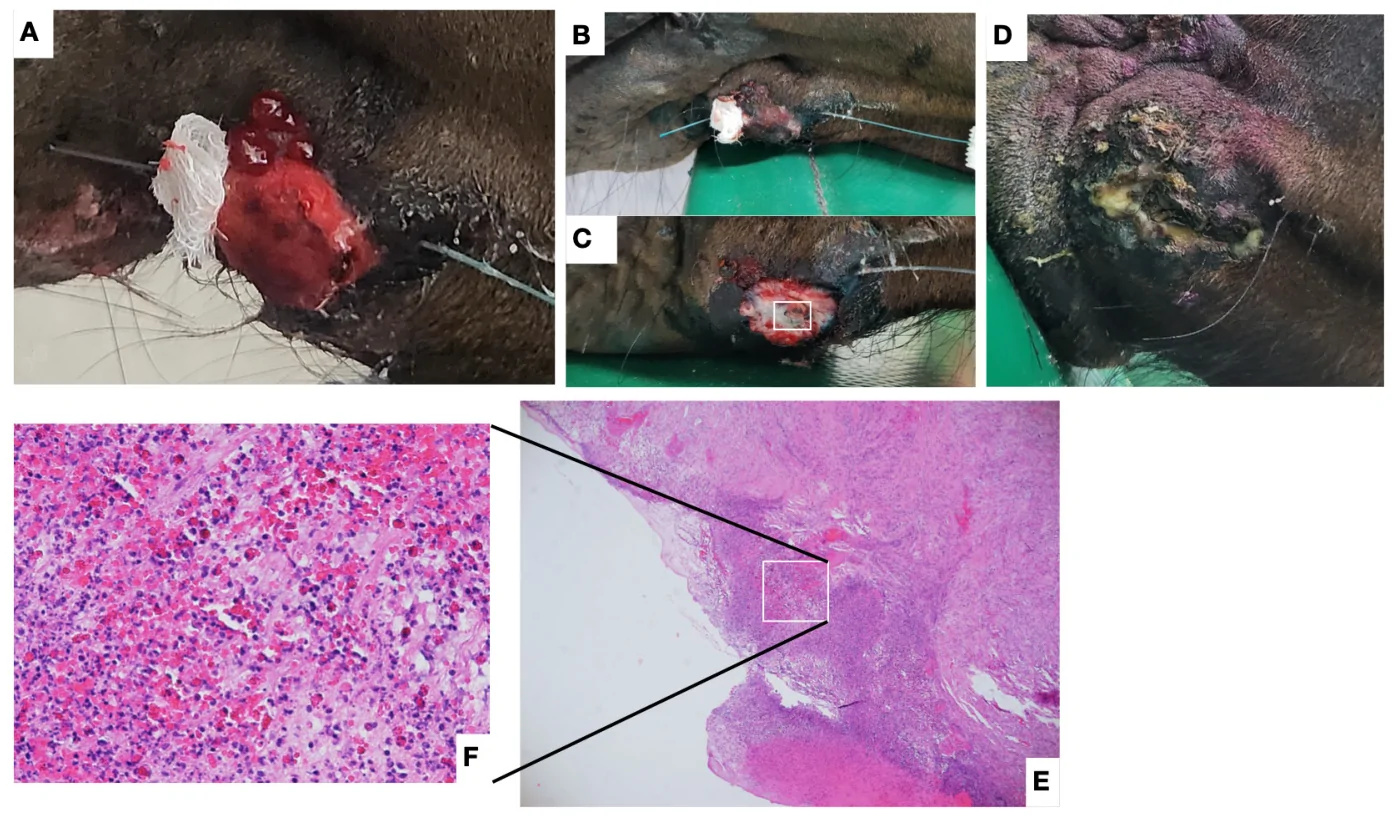
An OFM probe is introduced into the base of a sarcoid in Horse 2 (**A**). After perfusion (including dye; (**B**)) the surface of the tumour is trimmed to confirm the presence of the perfusant’s dye (**C**, **white rectangle**). At 4 days post perfusion, partial tumour oncosis is observed (**D**). Histology (H&E) shows a treatment-dependent recruitment of polymorphonucleated cells and vascular disruption at the base of the trimmed tumour mass ((**E**) 4×; (**F**) 40×).

Horse 2 (8-year-old Australian Stock horse mare)—One of two fibroblastic sarcoids (2 × 1.5 × 1 cm; T_Vol_ 1.5 cm^3^) underwent 7 h of OFM (0.04 mg/mL TT) using a single probe with a 1.5 cm long fluid exchange window. The other, distanced 15 cm, received a TT eluting implant. OFM was performed with blue dye added to the perfusate. Upon completion of OFM, the tumour was trimmed to the level of the surrounding skin, the exposed area inspected for discoloration and the resected tissue submitted for histopathology (hematoxylin and eosin, H&E, staining, Figure 3).

**Figure 4:**
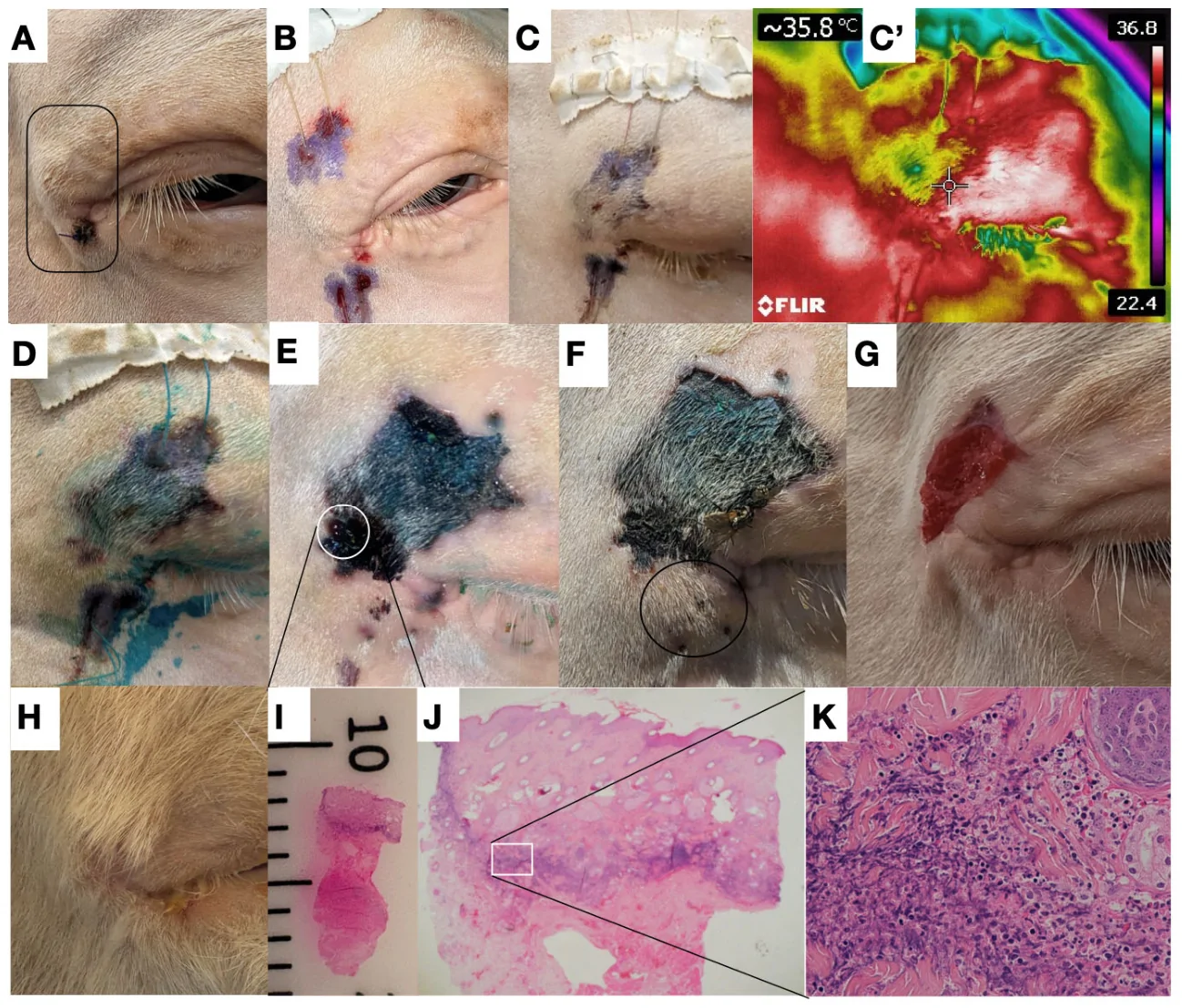
Horse 3 with squamous cell carcinoma in the upper eyelid and lateral canthus of the eye (circular highlight, (**A**)). Two perfusion probes in place (**B**), after perfusion on Day 1 (**C**) when skin temperature reduction (**C’**) and oncosis (**C**) is first noted and after gradually progressing (Day 2, (**D**); Day 3, (**E**)) to result in tissue separation (Day 4, (**F**)); treated tissue has sloughed (Day 25, (**G**)), and 4 months later, the external tumour mass was not apparent (**H**). Tissue associated with the probes’ “closed” portion remained viable ((**F**), black circle). A skin biopsy performed on the third day (white circle, (**E**)) confirms the treatment effect and marked influx of polymorphonucleated cells (0×, (**I**); 1.25×, (**J**); 40×, (**K**)).

Horse 3 (8-year-old Quarter horse mare)—A squamous cell carcinoma (1.5 × 2.5 × 1.4 cm; T_Vol_ 2.63 cm^3^) extending proximal from the lateral canthus of the right eye to become adherent with the periorbital rim was perfused (0.04 mg/mL TT) on two consecutive days for 7.5 and 8 h respectively using two probes, one with a 3 cm and one with a 1.5 cm perfusion window. On the second day, blue dye was added to the perfusate. Upon completion a 6 mm skin punch biopsy, 1 cm lateral from the position of the lateral probe, was obtained and submitted for histopathology (hematoxylin and eosin, H&E, staining, Figure 4).

**Figure 5:**
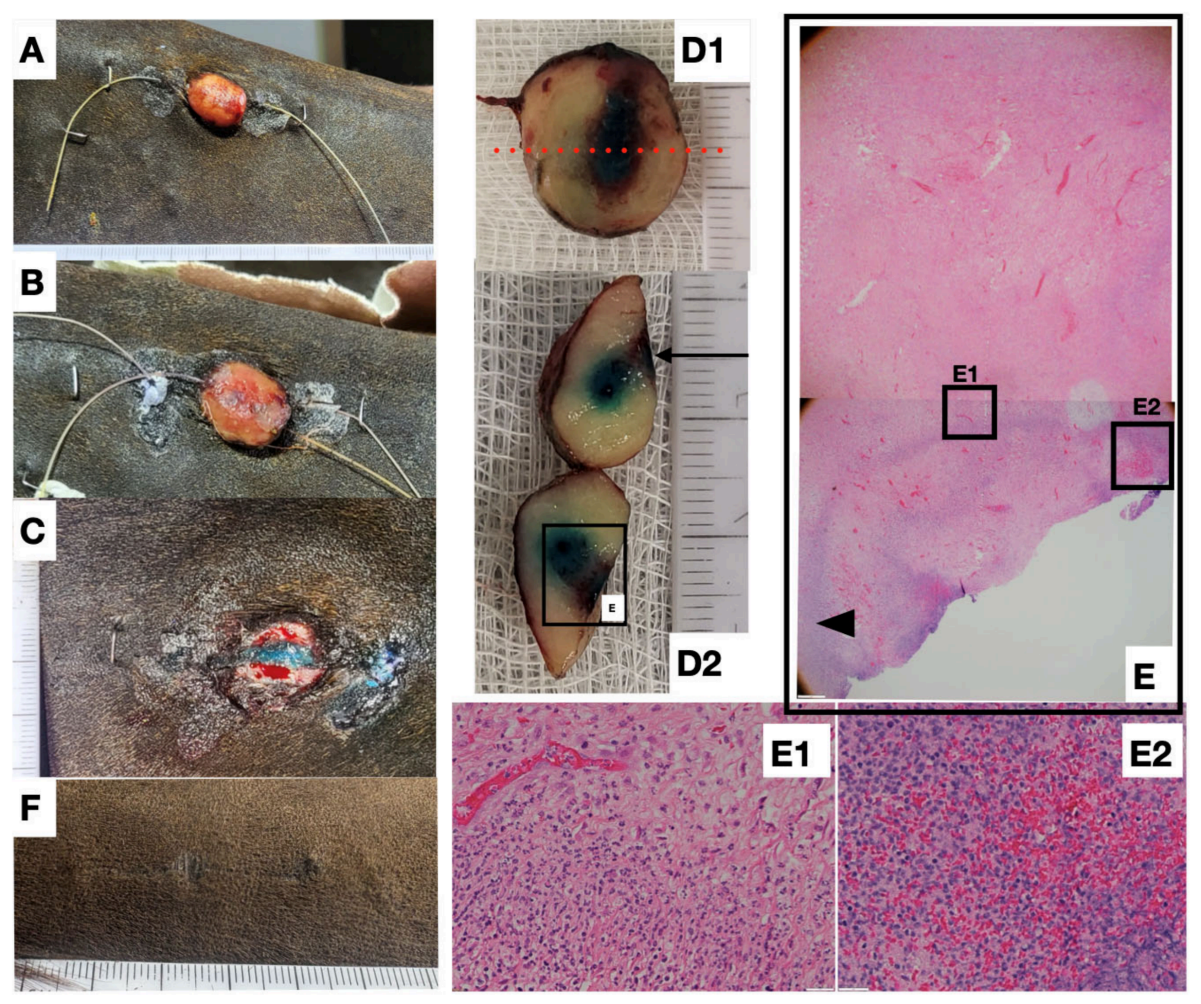
Horse 4 with fibroblastic sarcoid. Treatment site after perfusion on Day 1 (**A**) and Day 2 (**B**). After Day 3 perfusion, the tumour is resected above its base probe, exposing tissues participating in fluid exchange (**C**); base view of the resected tumour bulk (**D1**). The dotted line indicates the plane of sectioning to arrive at the image in (**D2**) and the orientation of histology sections (H&E, (**E**); 1.25×). Polymorphocucleated cell migration outlines the perfusion region of the base probe on the third day of treatment ((**E**), arrowhead). The treatment effect (haemorrhagic necrosis/oncosis and inflammatory cell recruitment) is clearly visible at the base of the tumour and exceeded the area of fluid exchange highlighted by the dye stain (arrow, (**D2**); highlight (**E1**,**E2**) at 40×). (**F**) Treatment site at last follow-up 497 days post treatment.

Horse 4 (10-year-old Standardbred mare)—A fibroblastic sarcoid (2 × 1.6 × 1.2 cm; T_Vol_ 1.92 cm^3^) was perfused on three consecutive days utilizing one tumour base probe on Day 1 and two probes (one through the base and centre of the tumour) on Day 2 and Day 3. The perfusion window measured 3 cm for the base and 1 cm for the centre probe. The base probe was perfused for 8 h utilizing a TT concentration of 0.01 mg/mL on Day 1, 0.02 mg/mL on Day 2 and 0.04 mg/mL on Day 3. The center probe was perfused for 5 h on Day 2 and 8 h on Day 3 utilizing 0.02 mg/mL and 0.04 mg/mL solutions respectively. Perfusions on Day 3 were performed with blue dye added. Upon completion of OFM, the tumour was trimmed to the level of the surrounding skin, discoloration in tumour tissue evaluated and tissue submitted for histopathology (hematoxylin and eosin, H&E, staining, Figure 5).

## 3. Results

In all applications of OFM with TT, drug induced immune responses were considered as expected and well controlled under the trial’s treatment protocol. No adverse events were observed, except for one probe requiring replacement (Horse 4; see below). Perfusion pumps were well tolerated, and none of the patients showed aversive behaviour leading to rubbing or interference with the system. Table 1 summarizes outcomes in the context of the relevant materials and methods.

Horse 1 (Figure 2): In this first use of the methodology, the OFM probe was successfully maintained and operated for 7 h. The horse’s perfusion set-up (Figure 2A), tissue appearance at termination of perfusion (Figure 2B), and after 5 days (Figure 2C) are illustrated. Following perfusion, the blue indicator dye was seen in the ventral (1, Figure 2D) but not the dorsal (2, Figure 2D) biopsy sample and the dark appearance of its sampling site was consistent with early TT-associated tissue changes (black arrow). Five days later, implant-induced oncosis and tissue slough was clearly apparent. Tissue demarcation extended into the pump-associated treatment area and oncosis included the ventral but not the dorsal sampling site (white box). An adjacent therapeutic implant (white star) is highlighted for orientation and to illustrate the expected associated oncosis pattern. Incomplete response to TT therapies required conventional therapies (electrochemotherapy) to be commenced 3 months from first treatment. The horse remains tumour-free 577 days from first treatment.

Horse 2 (Figure 3): A tumour deep probe was successfully perfused for 7 h. The probe was positioned with its distal end threaded through gauze and glued to the skin surface to stabilize the construct (Figure 3A). After perfusion (including dye; Figure 3B), the surface of the tumour was trimmed to confirm the presence of the perfusant’s dye (Figure 3C, white rectangle). At 4 days post perfusion, partial tumour oncosis following the probe’s previous location was observed (Figure 3D). Histology (H&E) showed a treatment-dependent recruitment of polymorphonucleated cells and vascular disruption at the base of the trimmed tumour mass in line with immune responses following TT treatment (Figure 3E,F) [15,16]. A TT eluting implant was inserted intratumourally on day 63 and the horse was tumour-free at last follow up 282 days later.

Horse 3 (Figure 4): The treatment site (squamous cell carcinoma) is shown pre- (Figure 4A,B) and post perfusion on Day 1 (Figure 4C) and Day 2 (Figure 4D). Oncosis and focal reduction in skin surface temperature were apparent from Day 1 (Figure 4C,C’). The tissue’s response became gradually more apparent (Day 3, Figure 4E) until tissue separation commenced (Day 4, Figure 4F). This process occurred at a distance between 5 and 8 mm from the nearest probe (Figure 4F) and resulted in an oncosis patch measuring approximately 2.6 × 2.2 cm. By Day 25 (Figure 4G) the treated tissue had sloughed, and on recheck 4 months later (Figure 4H), the external tumour mass was not apparent. It should be noted that only tissues exposed to the probes’ window of fluid exchange developed a response. The area immediately proximal to the probes’ exit sites and consistent with their “closed” portion remained viable (Figure 4F, black circle). The white circle shows the biopsy site performed on Day 3 (Figure 4E). The corresponding histology (H&E) confirmed the treatment effect and marked influx of polymorphonucleated cells approximately 1 cm from the probe location (Figure 4I–K). While the apparent tumour mass had been reduced, delayed disease progression and extension into ocular structures required exenteration of the patient’s globe 209 days following perfusion.

Horse 4 (Figure 5): The OFM system was successfully maintained and operated for the entire treatment duration. The base probe remained functional for 3 days, as shown by the unobstructed flow of perfusate on Day 3 (Supplemental Material, Video S1). The center probe had to be replaced once, as its unsupported position at tumour entry/exit resulted in breakage on Day 2. The treatment site is shown following perfusion solely through the tumour base (Day 1, Figure 5A) and through the base and center on Day 2 (Figure 5B). Reducing the tumour tissue after the second day of perfusing with two probes (Day 3) highlighted a 5–8 mm wide band of discoloration consistent with tissues participating in the fluid exchange at the tumour’s base (Figure 5C). The opposing surface in the corresponding tumour mass (Figure 5D1) showed less appreciable dye uptake, in contrast to its center and the area relating to the fluid exchange window of its probe (Figure 5D2). Judged by this pattern of dye dissemination, subtotal tumour exposure was achieved. TT-associated histopathologic changes (immune cell recruitment and vascular disruption) are visible including areas less clearly stained by the indicator dye (Figure 5E1,E2). On long-term follow-up, 497 days post treatment (Figure 5F) the treatment site remained tumour-free.

## 4. Discussion

Exploratory administration of TT using OFM produced local tissue responses consistent with the established effects of TT in equine patients [7]. Vascular disruption, inflammatory cell recruitment, oncosis, and subsequent tissue sloughing were observed, while treatment-associated inflammation was generally mild and comparable to that reported with other low-dose TT delivery strategies [10]. These findings provide proof of concept that OFM can achieve biologically relevant tissue exposure to TT and support its potential as a platform for controlled local delivery of tumour-targeted therapeutics. Given the nature of this scoping work and associated limitations, which are discussed below, the results do not support conclusions of therapeutic efficacy.

Importantly, OFM differs fundamentally from conventional intratumoural injection because the perfusate continuously traverses the treatment site before exiting the tissue. Consequently, the volume of TT solution passing through the system does not represent the amount of drug retained within the tumour, and the actual tissue dose could not be determined in this study. Even if complete retention of the perfusate were assumed, calculated TT exposure would have ranged from approximately 0.1 to 0.28 mg/cm^3^ of tumour tissue and would therefore have remained below previously recommended intratumoural doses [7]. Despite the flawed premise of equating TT delivery to perfusion metrics, this thought process was nevertheless utilized to select the experimental flow rate (9 μL/min) and principal perfusate concentration (0.04 mg/mL). In short assuming the worst-case scenario of blocked probes, those settings would have resulted in biologically relevant but low-dose administrations under the assumption of a minimum T_Vol_ of 1 cm^3^ and a maximum 8 h perfusion duration. Given that this scenario was not encountered, the development of characteristic TT-associated tissue responses in the face of continuous perfusate egress is of interest. Consequently, we speculate that sufficient exchange occurred between the perfusate and tumour interstitium to produce a pharmacologically relevant local effect at presumably much lower than previously reported dose rates.

The spatial distribution of these responses provides further evidence of controlled tissue exposure. Macroscopic oncosis generally corresponded closely to the location of the probes and, more specifically, to their open fluid exchange windows. This was particularly evident in Horse 3, in which tissue immediately adjacent to the closed portion of the probe remained viable, whereas tissue exposed to the exchange window underwent oncosis and subsequent sloughing. The resulting treatment area had a sharply demarcated, approximately rhomboid configuration, illustrating the potential of OFM to achieve predictable spatial distribution of a therapeutic agent. Histopathological evidence of vascular disruption and polymorphonuclear cell recruitment was also identified at distances from the probes and, in some instances, beyond areas visibly stained by the indicator dye. Together, these observations suggest that visible perfusate distribution may underestimate the extent of biologically relevant TT exposure.

Clinical responses varied between cases, as expected in this exploratory study. Of the three horses in which OFM with TT represented the principal treatment modality, one (Horse 4) achieved a sustained long-term response, with the treatment site remaining tumour-free 497 days after treatment. This outcome occurred despite dye distribution suggesting incomplete exposure of the tumour base. Conversely, histopathological changes extended beyond visibly stained tissue, potentially indicating either wider TT distribution than could be demonstrated macroscopically or amplification of the local response through the inflammatory and immunomodulatory effects of TT. The possibility that surgical reduction of the tumour following treatment contributed to resolution cannot be excluded but should be considered unlikely. Conventional surgical excision, removing all tissue that is perceived to be affected, is generally associated with a substantial risk of recurrence in equine sarcoids [17]. Given that tumour reductions were performed to expose perfusion effects and deliberately left macroscopically diseased tissue behind, this supports a contribution from TT exposure to the observed outcome.

The responses in Horses 2 and 3 also provide insight into factors likely to influence the efficacy of OFM-mediated drug delivery. In Horse 2, a single probe was positioned relatively deep within the tumour. The resulting oncosis formed a narrow tract corresponding to the probe position but was insufficient to induce complete tumour sloughing. This observation suggests that adequate coverage of the tumour base, rather than simply intratumoural probe placement, may be important for successful treatment. In Horse 3, OFM resulted in complete ablation of the externally apparent component of a periocular squamous cell carcinoma; however, deeper disease subsequently progressed and ultimately necessitated exenteration. Thus, local control of the perfused tissue should not be interpreted as control of tumour extending beyond the treatment field. Nevertheless, the sharply defined tissue response in this anatomically sensitive region illustrates a potentially important advantage of OFM: the ability to restrict therapeutic exposure where inadvertent delivery to adjacent structures may result in clinically important morbidity.

Several characteristics of OFM warrant further investigation before optimal treatment parameters can be defined. The system was operated at a flow rate of 9 µL/min, corresponding to the maximum setting used in the present study. We speculate that lower flow rates may increase residence time and facilitate exchange between the perfusate and interstitial compartment, potentially increasing the magnitude or spatial extent of tissue exposure. Similarly, probe number, orientation, exchange window length, and inter-probe spacing are likely to influence drug distribution. Based on the responses observed here, we propose an inter-probe distance of approximately 1 cm as a reasonable starting point. Fluid exchange is also likely to depend on tumour-specific characteristics, including tissue density, vascularity, interstitial pressure, and extracellular matrix composition. Controlled studies examining these variables alongside direct measurement of tissue TT concentrations will therefore be required to establish the pharmacokinetics and optimal fluid dynamics of OFM-mediated delivery.

OFM also provides opportunities beyond therapeutic administration. When operated in a push–pull configuration, the system permits simultaneous recovery of interstitial fluid and could therefore combine local drug delivery with temporal sampling of the tumour microenvironment. This may enable treatment exposure to be related directly to local pharmacokinetics, inflammatory mediators, biomarkers, and cellular responses. Such an approach could be particularly valuable for mechanistic studies of TT, for which vascular and immunological responses are integral to its antineoplastic activity.

Although the present study used TT as a model therapeutic agent, the broader relevance of OFM as a drug delivery platform warrants consideration. Continuous low-flow administration could potentially provide controlled local exposure to other antineoplastic agents. Application to conventional cytotoxic agents, including platinum-based compounds, would, however, require a closed perfusion system and appropriate safeguards against environmental exposure. The approach may be particularly suited to immunomodulatory therapies intended to modify the tumour microenvironment and enhance local anti-tumour immunity [18,19,20]. In this context, the ability to combine spatially controlled drug exposure with interstitial fluid sampling could provide both therapeutic and investigative advantages.

From a practical perspective, OFM was well tolerated in horses, and the system could be maintained during normal but stall-restricted patient activity. Probes remained functional for up to three consecutive days, although one centrally positioned probe required replacement following mechanical failure. These findings demonstrate the feasibility of prolonged OFM in a large-animal clinical setting while also identifying probe stabilisation as an important consideration. Application in smaller veterinary patients may be more challenging because of greater relative mobility and reduced capacity to protect external components, although intermittent perfusion under sedation with probes retained between treatment sessions may be feasible. Conversely, maintenance of comparable systems in human patients may present fewer practical constraints.

The principal limitations of this study are its exploratory case-series design, small and heterogeneous study population, variable treatment protocols, and concurrent therapeutic interventions in two horses. TT concentration, number and position of probes, duration of perfusion, and number of treatment days varied between cases, precluding meaningful assessment of dose–response relationships or clinical efficacy. Furthermore, indicator dye provided only a qualitative estimate of fluid distribution and cannot be assumed to represent TT concentrations within the tissue. The actual amount of TT transferred from the perfusate into the tumour interstitium was not measured. Accordingly, the present findings should be interpreted as evidence of technical feasibility and biological activity rather than therapeutic efficacy.

Nevertheless, the development of spatially defined vascular and inflammatory responses progressing to tissue oncosis demonstrates that biologically relevant exposure of tumour tissue to TT can be achieved using OFM. Further studies incorporating controlled treatment protocols, quantitative pharmacokinetic measurements, systematic histopathological assessment, and defined clinical endpoints are warranted to determine the relationship between OFM parameters, tissue drug exposure, and therapeutic response. Such studies will establish whether OFM can progress from an experimental delivery method to a clinically useful platform for precise, sustained intratumoural therapy.

## 5. Conclusions

Open flow microperfusion of equine cutaneous tumours is technically feasible and results in distinct and localized biologic responses when perfusing with tigilanol tiglate. The findings justify ongoing efforts in a wider population with more controlled experimental methods to assess the therapeutic utility of this platform for local delivery of tumour-targeted therapies.

## Figures and Tables

**Table 1:** Summary of treatment details and outcomes.

Horse	Tumour Type	Tumour Measurements	Probe/Window	Duration (h)	Day	TT Conc. in Perfusate (mg/mL)	Total TT Perfused	Biological Effect	Outcome with TT-OFM Therapy	Comments
**Horse 1**	Sarcoid	n/a	Probe 1 (intradermal)/0.5 mm	7	D1	0.04	0.152 mg	Presence of dye and gross pathological changes consistent with TT effects	Incomplete response (tumour persistence)	Treatment site adjacent to primary tumour receiving concurrent TT therapies; Full response with alternate therapies
**Horse 2**	Sarcoid	2 × 1.5 × 1 cm T_Vol_ 1.5 cm^3^	Probe 1 (tumour base)/1.5 cm	7	D1	0.04	0.152 mg 0.1 mg/cm^3^	Presence of dye and partial tumour slough along perfusion probe axis, Histopathologic changes consistent with TT exposure	Incomplete response (tumour persistence)	Tumour trimmed post perfusion; Autotransplantation; Full response with continued TT therapies
**Horse 3**	SCC	1.5 × 2.5 × 1.4 cm T_Vol_ 2.63 cm^3^	Probe 1 (tumour base)/1.5 cm	7.5	D1	0.04	0.668 mg 0.254 mg/cm^3^	Oncosis and tissue slough of target area; Histopathologic changes consistent with TT exposure	Incomplete response (deep tumour recurrence)	Full response with subsequent surgical treatments
				8	D2	0.04				
			Probe 2 (tumour base)/3 cm	7.5	D1	0.04				
				8	D2	0.04				
**Horse 4**	Sarcoid	2 × 1.6 × 1.2 cm T_Vol_ 1.92 cm^3^	Probe 1 (tumour base)/3 cm	8	D1	0.01	0.53 mg 0.276 mg/cm^3^	Presence of dye; Oncosis and tissue slough of target area; Histopathologic changes consistent with TT exposure	Complete response (497 days follow up)	Tumour trimmed post perfusion; Probe 2 replaced on D3 due to breakage
				8	D2	0.02				
				8	D3	0.04				
			Probe 2 (tumour center)/1 cm	5	D2	0.02				
				8	D3	0.04				

## Data Availability

All relevant data is included in this manuscript.

## Supplementary Materials

The following supporting information can be downloaded at https://www.mdpi.com/article/10.3390/ani16193090/s1, Video S1: Perfusate flow on Day 3 in Horse 4.
